# Fish Consumption and Colorectal Neoplasms among Lynch Syndrome Carriers

**DOI:** 10.1016/j.tjnut.2026.101670

**Published:** 2026-06-17

**Authors:** Marjolein T Bulbaai, Evertine Wesselink, Tanya M Bisseling, Jan Jacob Koornstra, Monique E van Leerdam, Loïc Le Marchand, Polly A Newcomb, N Jewel Samadder, Hanna JM Schep-Wijnveen, Ellen Kampman, Fränzel J van Duijnhoven

**Affiliations:** 1 Division of Human Nutrition and Health, Wageningen University & Research, Wageningen, The Netherlands; 2 Department of Gastroenterology, Radboud University Medical Center, Nijmegen, The Netherlands; 3 Department of Gastroenterology and Hepatology, University Medical Center Groningen, University of Groningen, Groningen, The Netherlands; 4 Department of Gastroenterology and Hepatology, Leiden University Medical Center, Leiden, The Netherlands; 5 The Netherlands Foundation for the Detection of Hereditary Tumors, Leiden, The Netherlands; 6 Department of Gastrointestinal Oncology, The Netherlands Cancer Institute, Amsterdam, The Netherlands; 7 Population Sciences in the Pacific (Epidemiology), University of Hawaii Cancer Center, Honolulu, HI, United States; 8 Public Health Sciences Division, Fred Hutchinson Cancer Center, Seattle, WA, United States; 9 Department of Epidemiology, University of Washington, Seattle, WA, United States; 10 Division of Gastroenterology and Hepatology, Mayo Clinic, Scottsdale, AZ, United States

**Keywords:** fish intake, colorectal neoplasm, Lynch syndrome, mismatch repair deficient, cohort study

## Abstract

**Background:**

Fish intake may exert antineoplastic effects, potentially through omega-3 fatty acids. However, its role in the development of colorectal neoplasms (CRNs) among individuals with Lynch syndrome (LS), a genetic predisposition to colorectal cancer, remains unclear.

**Objectives:**

The aim of this study was to examine the association between fish intake and CRN risk among LS carriers, overall and across subgroups defined by nonsteroidal anti-inflammatory drug/aspirin use, sex, geographical region, CRN history, and pathogenic variant type, and to examine associations between fish types and CRN risk.

**Methods:**

Data from 1816 confirmed LS carriers in the GEOLynch cohort (The Netherlands) and the Colon Cancer Family Registry (Australia, Canada, and the United States) were used. Information on fish intake was collected at inclusion using food frequency questionnaires. CRNs included adenoma, serrated lesions, and colorectal cancer. Multivariable Cox proportional hazards models estimated hazard ratios (HRs) and 95% confidence intervals (CIs) across sex-specific quartiles and per 10 g/d of total fish intake. Associations for specific fish types were assessed per 5 g/d increase.

**Results:**

During a median follow-up time of 7.0 y (IQR range: 2.8–13.6), 742 (40.9%) participants developed a CRN. The CRN risk was similar in individuals in the highest quartile of fish intake compared with the lowest quartile (HR 1.03; 95% CI: 0.81, 1.32). No association was observed for every 10 g/d increment in fish intake (HR: 1.0; 95% CI: 0.95, 1.05) and across subgroups. However, a 5 g/d increase in canned fish appeared to increase the risk of CRNs (HR: 1.06; 95% CI: 1.01, 1.10).

**Conclusions:**

Total fish intake does not appear to influence the development of CRNs in individuals with LS, whereas the influence of canned fish intake on CRN risk should be further investigated.

## Introduction

Lynch syndrome (LS) is one of the most common hereditary forms of colorectal cancer (CRC), caused by a germline pathogenic variant in one of the DNA mismatch repair (MMR) genes—*MLH1*, *MSH2*, *MSH6*, or *PMS2*—or by a deletion in the *EpCAM* gene [[1], [2], [3]]. Because of this genetic predisposition, individuals with LS experience an accelerated colorectal adenoma–carcinoma sequence [3] or may develop CRC directly by bypassing the conventional adenomatous pathway [4]. As a result, they have a significantly higher lifetime risk of developing CRC [5] compared with the general population [6]. Observed variability in CRC risk among the same LS-affected families indicates that lifestyle and dietary factors might also contribute to the development of colorectal neoplasms (CRNs) [[7], [8], [9]], which include CRC and its precursor lesions, adenoma and serrated lesions (SLs) [10].

Long-chain omega (ω)-3 polyunsaturated fatty acids, mainly consumed through fish intake, may exert antineoplastic effects through their anti-inflammatory, proapoptotic, antiproliferative, and antiangiogenic properties [11]. In the general population, epidemiological studies on total fish intake and CRC and adenoma risk have been inconsistent. Although a recent meta-analysis observed a modest inverse association between total fish intake and sporadic CRC [summary risk ratio: 0.94; 95% confidence interval (CI): 0.89, 0.99] [12], another meta-analysis observed no association with sporadic adenomas (summary risk estimate: 0.98; 95% CI: 0.18, 1.18) [13]. However, little is known about the relationship between fish intake and CRN risk in individuals with LS. One case-control study among confirmed and suspected LS carriers observed no association between total fish intake and CRN risk [14]. This study was limited by its small sample size and retrospective design. Moreover, it did not evaluate the type of fish consumed, nor the potential modifying roles of nonsteroidal anti-inflammatory drugs/aspirin (NSAIDs/aspirin) use, sex hormones, geographical variation, a previous CRN diagnosis, or the pathogenic MMR gene. Because the ω-3 content differs substantially by type of fish, with fatty fish containing higher levels than lean fish [15], it is essential to investigate whether these associations vary according to the type of fish consumed. ω-3 fatty acids are believed to exert additive or synergistic antineoplastic effects with NSAIDs/aspirin through the cyclooxygenase pathway [16]. In addition, sex-specific differences in fatty acid metabolism, potentially due to estrogen [11], may further modify the association between fish intake and CRN risk. Geographical variation in fish consumption and preparation methods, as well as aquatic conditions such as water temperature, salinity, and depth, may influence the ω-3 content [11] and, therefore, the association in different geographical regions. Furthermore, a CRN history may modify the association, as shown in prior research from our group on the association between BMI and adenoma [17]. The pathogenic MMR gene involved may also play a role, because the adenoma–carcinoma pathway differs depending on the pathogenic MMR gene [3,4,18]. These potential modifying factors highlight the complexity and underscore the need for stratified analyses to better understand the role of fish intake in CRN risk among LS carriers.

Given the inconclusive results and the limited research specifically among LS carriers, the primary objective of this study was to examine the association between fish intake and CRN development in individuals with confirmed LS in a multicenter, international study. Secondary objectives were to assess whether the association varies across subgroups defined by NSAID/aspirin use, sex, geographical region, CRN history, and pathogenic MMR gene. Lastly, this is the first study to explore associations between different types of fish and CRN risk in LS carriers, providing insights into how various fish types may be related to CRN risk.

## Methods

### Study population

We used data from 2 previously described cohort studies, the GEOLynch “Genetic, Environmental and Other factors that influence tumor risk in persons with Lynch syndrome” (clinicaltrials.gov identifier NCT03 303833) and the CCFR “Colon Cancer Family Registry Cohort” [19,20].

**FIGURE 1:**
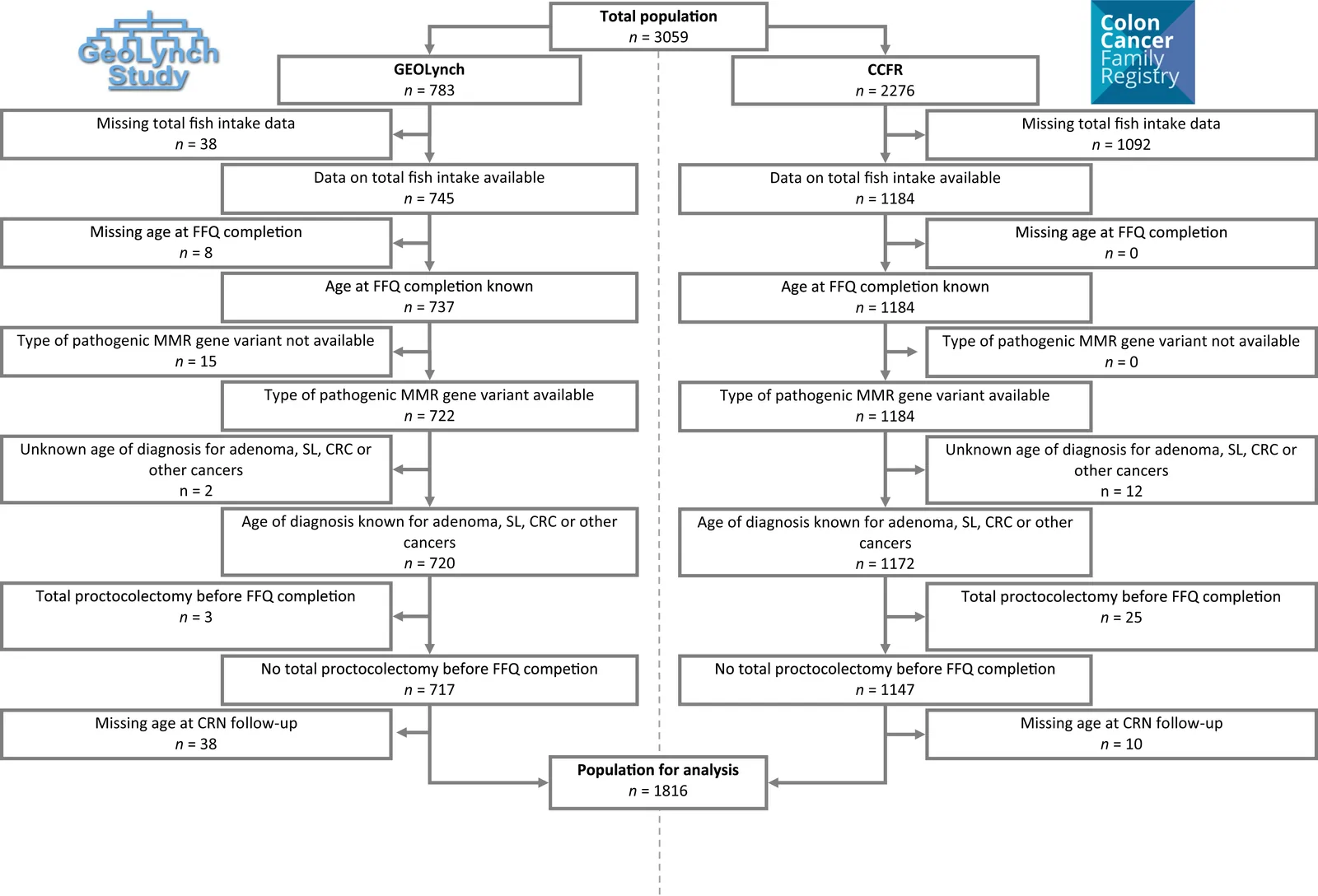
Flowchart representing participant selection. CCFR, Colon Cancer Family Registry; CRC, colorectal cancer; CRN, colorectal neoplasm; FFQ, food frequency questionnaire; SL, serrated lesion; GEOLynch, Genetic, Environmental and Other factors that influence tumor risk in persons with Lynch Syndrome; MMR, mismatch repair.

Participants for the GEOLynch study were actively recruited in 2 phases: phase I (2006–2008) and phase II (2012–2017) through the Netherlands Foundation for the Detection of Hereditary Tumors (Leiden), Radboud University Medical Center (Nijmegen), and University Medical Center Groningen (Groningen) [21]. Since 2012, participants have also been passively recruited through the Lynch Polyposis Society [21]. Eligible individuals were Dutch-speaking adults (18–80 y) with a confirmed germline pathogenic variant associated with LS (*n* = 783, Figure 1). Exclusion criteria included cognitive impairment or a diagnosis of inflammatory bowel disease.

The CCFR cohort, previously described in detail [19,20], is an international consortium comprising 6 research centers located in Australia, Canada, and the United States. Participants were enrolled through population-based and clinic-based recruitment methods across 3 phases: phase I (1998–2002), phase II (2003–2007), and phase III (2008–2012). Population-based recruitment identified probands diagnosed with CRC via cancer registries. In contrast, clinic-based recruitment included individuals with or without a personal history of cancer attending genetic or familial cancer clinics. Only individuals with LS with a confirmed germline pathogenic variant were included in this study (*n* = 2276, Figure 1).

Both studies were approved by their local medical ethics review committees. GEOLynch was approved by the Committee on Research Involving Human Subjects, region Arnhem-Nijmegen (CMO-nr: 2005/283). The CCFR study protocols were approved by the Human Subjects/Ethics Boards at each participating site: Cleveland Clinic Institutional Review Board (nr: 2913), University of Hawaii Human Studies Program (nr: 10057), University of California, San Francisco Human Research Protection Program Institutional Review Board (nr: 17-23404), Mount Sinai Hospital Research Ethics Board (nr: 01-0347-U), and University of Melbourne Health Sciences Human Ethics Sub-Committee Ethics (nr: 13094). All participants provided written informed consent.

### Population for analysis

Participants were excluded in the case of missing information related to: *1*) total fish intake (*n* = 1130), *2*) the age at food frequency questionnaire (FFQ) completion (*n* = 8), *3*) specific pathogenic MMR gene variant (*n* = 15), or *4*) the age at diagnosis for the first adenoma, SL, CRC, or other cancer (*n* = 14). In addition, participants who underwent total proctocolectomy before completing the FFQ (*n* = 28) and those with no age at the end of follow-up time (*n* = 48) were excluded (Figure 1). The final analysis population consisted of 1816 participants out of a total of 3059.

### Assessment of fish intake

Total fish intake includes all types of fresh (raw, baked, boiled, broiled), canned, salted, dried, or fried fish, covering both fatty and nonfatty varieties (including shellfish). Fish intake was assessed using country-specific semiquantitative FFQs developed by the Division of Human Nutrition and Health at Wageningen University (The Netherlands) [22,23] for participants in the Netherlands, the Epidemiology Program Cancer Research of Hawai’i, the University of Hawai’i (United States) [24,25] for participants in the United States and Canada, and the Cancer Council Victoria (Australia) [26] for participants in Australia. The FFQs inquired about the type of fish, frequency, number of portions, and portion sizes at 1 mo (in the Netherlands), 1 y (in Australia), and 2 y (in the United States) before recruitment. This information was used to calculate the mean daily fish intake (grams) for each fish type. Total fish intake was calculated by summing the mean daily intake of all fish types.

### Assessment of CRNs

CRNs were defined as adenomas, SLs (including hyperplastic polyps, sessile SLs, or traditional serrated adenomas), or CRCs [10]. Information on CRNs that occurred before or during the follow-up period of the GEOLynch study was collected through medical and pathology records up to 2024. For CCFR participants, CRN data were compiled from medical and pathology records, cancer registry reports, death certificates, and self-reports or reports from relatives at baseline, as well as at subsequent follow-ups. These data were supplemented by linkage to cancer registries, each following study center–specific protocols.

### Collection and assessment of covariates

Data on demographics (age, sex, and educational level), lifestyle factors (smoking habits, physical activity levels, and NSAID/aspirin use), anthropometric measurements (height and weight), and dietary intake other than fish intake were collected during the recruitment phase through standardized questionnaires. These questionnaires were either self-administered (GEOLynch) or conducted through personal interviews, telephone interviews, or mail (CCFR). Information about participants’ colorectal surgeries that occurred before and throughout the follow-up period was obtained from medical and pathology records, self-reports (GEOLynch/CCFR), and cancer registries (CCFR).

### Data analysis

Baseline characteristics were presented as mean (SD) for normally distributed continuous variables, median (Q1, Q3) for nonnormally distributed continuous variables, and counts (%) for categorical variables based on the total population and across sex-specific quartiles of total fish intake (Table 1). Hazard ratios (HRs) and corresponding 95% CIs were estimated across sex-specific quartiles, with the lowest quartile as reference, and per 10 g/d increment in total fish intake using Cox proportional hazards regression. The person-time began at the age when the FFQ was completed and ended with one of the following events: CRN diagnosis, cancer diagnosis excluding nonmelanoma skin cancers, total proctocolectomy, death, last contact (CCFR), loss to follow-up (GEOLynch), clinical trial enrolment (GEOLynch), or last colonoscopy, whichever occurred first. Proportional hazards assumptions were assessed using Schoenfeld residuals.

**TABLE 1:** Characteristics of the overall population at recruitment and stratified by quartiles of fish intake

	Total population	Quartile 1	Quartile 2	Quartile 3	Quartile 4
		Males: <4.5 g/d	4.5–<12.9	12.9–<21.2	≥21.2
		Females: <4.7 g/d	4.7–<11.4	11.4–<19.1	≥19.1
	1816	452	455	441	468
Demographic characteristics					
Age (y) at questionnaire completion, median [Q1, Q3]	48.8 [38.5, 58.3]	45.0 [33.6, 56.4]	48.1 [38.9, 57.7]	49.7 [40.9, 59.0]	51.2 [40.3, 59.2]
Sex (female), *n* (%)	1057 (58.2)	267 (59.1)	261 (57.4)	258 (58.5)	271 (57.9)
Education level, *n* (%)					
Low	517 (28.8)	141 (31.6)	130 (28.8)	117 (26.7)	129 (28.0)
Medium	796 (44.3)	213 (47.8)	206 (45.6)	192 (43.8)	185 (40.1)
High	484 (26.9)	92 (20.6)	116 (25.7)	129 (29.5)	147 (31.9)
Unknown, *n*	19	6	3	3	7
Race, White, *n* (%)	1743 (96.0)	440 (97.8)	440 (97.1)	427 (97.0)	436 (94.0)
Unknown, *n*	9	2	2	1	4
Geographical region of recruitment, *n* (%)					
Australia (CCFR)	874 (48.1)	284 (62.8)	215 (47.3)	154 (34.9)	221 (47.2)
Canada (CCFR)	160 (8.8)	28 (6.2)	58 (12.7)	33 (7.5)	41 (8.8)
Netherlands (GEOLynch)	679 (37.4)	124 (27.4)	155 (34.1)	230 (52.2)	170 (36.3)
United States (CCFR)	103 (5.7)	16 (3.5)	27 (5.9)	24 (5.4)	36 (7.7)
Clinical characteristics					
Follow-up time (y), median [Q1, Q3]	7.0 [2.8, 13.6]	9.0 [3.4, 14.5]	7.5 [3.4, 13.5]	5.7 [2.2, 12.8]	6.5 [2.3, 12.9]
Age (y) at the end of follow-up^1^, median [Q1, Q3]	54.5 [44.2, 66.1]	54.5 [44.2, 65.0]	56.9 [47.0, 66.1]	57.5 [48.6, 65.8]	57.5 [48.0, 67.3]
Mutated MMR gene, *n* (%)					
*EpCAM*	30 (1.7)	9 (2.0)	9 (2.0)	3 (0.7)	9 (1.9)
*MLH1*	692 (38.1)	183 (40.5)	180 (39.6)	171 (38.8)	158 (33.8)
*MSH2*	772 (42.5)	192 (42.5)	187 (41.1)	180 (40.8)	213 (45.5)
*MSH6*	240 (13.2)	57 (12.6)	58 (12.7)	59 (13.4)	66 (14.1)
*PMS2*	82 (4.5)	11 (2.4)	21 (4.6)	28 (6.3)	22 (4.7)
Individuals with a history of CRN diagnosis^2^, *n* (%)	961 (52.9)	207 (45.8)	243 (53.4)	243 (55.1)	267 (57.1)
Number of colonoscopies, median [Q1, Q3]	11.0 [7.0, 16.0]	11.0 [6.0, 15.0]	11.0 [7.0, 17.0]	11.5 [8.0, 16.0]	10.0 [6.0, 15.0]
Unknown, n	15	5	4	3	3
Dietary factors					
Total fish intake (g/d), median [Q1, Q3]	11.9 [4.6, 20.0]	1.5 [0.0, 3.0]	9.2 [6.9, 10.6]	16.0 [14.2, 17.0]	34.5 [24.4, 45.4]
Total fish intake ≥ 50 g/d, *n* (%)	0 (0.0)	0 (0.0)	0 (0.0)	0 (0.0)	96 (5.3)
Fatty fish intake (g/d)^3^, median [Q1, Q3]	3.7 [0.0, 7.3]	0.0 [0.0, 0.0]	2.5 [0.0, 4.3]	4.6 [2.9, 6.8]	10.4 [5.6, 15.4]
Lean fish intake (g/d)^3^, median [Q1, Q3]	4.8 [0.0, 9.4]	0.0 [0.0, 0.0]	3.4 [0.2, 5.3]	5.5 [3.3, 8.4]	15.4 [7.8, 23.0]
Fried fish intake (g/d)^4^, median [Q1, Q3]	2.6 [0.0, 7.2]	1.6 [0.0, 5.5]	2.8 [0.0, 5.8]	2.9 [0.0, 7.3]	2.9 [0.0, 10.3]
Canned fish intake (g/d)^5^, median [Q1, Q3]	5.0 [1.4, 12.6]	2.2 [0.0, 6.9]	5.0 [1.8, 9.5]	7.6 [3.0, 12.0]	12.6 [2.4, 26.9]
Alcohol intake (g/d), median [Q1, Q3]	5.5 [0.6, 17.3]	3.3 [0.3, 13.4]	6.1 [0.7, 17.5]	6.4 [1.0, 17.4]	8.1 [0.5, 19.6]
Fruit and vegetable intake (g/d), median [Q1, Q3]	318.3 [213.3, 450.5]	253.8 [180.4, 377.1]	284.8 [199.5, 410.6]	331.0 [221.7, 452.7]	395.8 [288.3, 564.9]
Fiber intake (g/d), median [Q1, Q3]	21.6 [17.0, 28.1]	19.5 [14.9, 25.5]	20.0 [15.9, 25.9]	23.3 [17.8, 28.7]	24.8 [19.5, 31.6]
Red and processed meat intake, median [Q1, Q3]	71.9 [42.4, 110.6]	72.9 [41.1, 115.7]	70.4 [41.9, 104.9]	68.7 [47.1, 100.5]	74.9 [37.8, 119.1]
Energy intake (kcal/d), median [Q1, Q3]	1941.8 [1523.8, 2523.0]	1820.8 [1429.3, 2325.1]	1836.9 [1429.1, 2436.6]	2021.6 [1604.8, 2558.0]	2142.9 [1715.4, 2795.7]
Lifestyle factors					
BMI 2 y before baseline questionnaire completion (kg/m^2^), median [Q1, Q3]	24.9 [22.4, 28.1]	25.3 [22.4, 28.4]	24.5 [22.2, 27.4]	25.0 [22.7, 27.7]	24.9 [22.4, 28.4]
Unknown, *n*	47	13	8	11	15
Smoking status, *n* (%)					
Never	867 (47.7)	216 (47.8)	232 (51.0)	201 (45.6)	218 (46.6)
Former^6^	656 (36.1)	153 (33.8)	152 (33.4)	170 (38.5)	181 (38.7)
Current^7^	293 (16.1)	83 (18.4)	71 (15.6)	70 (15.9)	69 (14.7)
Physical activity level, *n* (%)^8^					
Low	551 (32.1)	134 (31.3)	134 (31.8)	144 (34.7)	139 (30.9)
Medium	581 (33.9)	159 (37.1)	143 (34.0)	139 (33.5)	140 (31.1)
High	582 (34.0)	135 (31.5)	144 (34.2)	132 (31.8)	171 (38.0)
Unknown, *n*	102	24	34	26	18
NSAIDs/aspirin use, *n* (%)					
No use	1270 (71.6)	329 (74.1)	318 (72.6)	304 (70.5)	319 (69.2)
Less than once per day	210 (11.8)	44 (9.7)	47 (10.3)	63 (14.2)	56 (12.0)
At least once per day	294 (16.6)	71 (16.0)	73 (16.7)	64 (14.8)	86 (18.7)
Unknown, *n*	42	8	17	10	7

Abbreviations: BMI, body mass index; CCFR, Colon Cancer Family Registry; CRN, colorectal neoplasm; FFQ, food frequency questionnaire; GEOLynch, Genetic, Environmental and Other factors that influence tumor risk in persons with Lynch Syndrome; MMR, mismatch repair; NSAID, nonsteroidal anti-inflammatory drug; Q, quartile.

1 The age at which one of the following events occurred first: first diagnosed colorectal neoplasm, cancer excluding nonmelanoma skin cancers, total proctocolectomy, death, last contact, loss to follow-up, last colonoscopy, clinical trial enrolment, or last age known to be alive.

2 A history of CRN was defined as a CRN diagnosis occurring either before the completion of the FFQ.

3 Information was only available for individuals from The Netherlands.

4 Information was available for individuals from Australia and The Netherlands.

5 Information was only available for individuals from Australia.

6 Former smokers are defined as individuals who have a history of smoking for ≥3 mo.

7 Current smokers are defined as persons who have smoked for ≥3 mo at the time of the questionnaire.

8 Cohort-specific tertiles of physical activity (GEOLynch: total index, CCFR: metabolic equivalent of task score).

Restricted cubic splines, with no fish intake as the reference, were used to assess a potential nonlinear association between total fish intake and CRN risk. The knots were positioned at the 10th, 50th, and 90th percentiles of total fish intake based on Akaike’s information criteria and model parsimony. Confounding variables were identified through a directed acyclic graph [27]. The adjustment set included age at FFQ completion, sex, BMI, smoking status, educational level, and the number of colonoscopies, alcohol intake, fruit and vegetable intake, red and processed meat intake, physical activity, NSAID/aspirin intake, and geographical region. Variables that violated the proportional hazards assumption were included in the Cox proportional hazard model as a stratum. Robust sandwich variance estimates were used to account for the correlation among family members by clustering on the family number.

Subgroup analyses were performed by NSAID/aspirin use (none, <1/d, and ≥1/d), sex (male and female), geographical region (Australia, Canada, the Netherlands, and the United States), CRN history (no history and history of CRN), pathogenic MMR gene type (*MLH1*, *MSH2/EpCAM*, *MSH6*, and *PMS2*), and study cohort (CCFR and GEOLynch) through stratification by these factors and by calculating the *P* value for heterogeneity using the inverse variance method.

In addition, we separately explored the association between several types of fish (fatty fish, lean fish, fried fish, and canned fish) and CRN risk using restricted cubic splines, with no intake as the reference, and we analyzed fish intake as a continuous variable per 5 g/d increment.

Several sensitivity analyses were conducted. First, we excluded individuals diagnosed with a CRN within 100 d after completing the FFQ to assess potential reverse causality. Second, we evaluated the separate outcomes adenoma, CRC, or advanced CRN. Advanced CRNs were defined as advanced adenoma (diameter ≥10 mm, high-grade dysplasia, or ≥25% villous histology), advanced SL (any SL with a diameter ≥10 mm or dysplasia), or CRC [28,29]. Third, a weighted Cox proportional hazards analysis was performed to account for potential ascertainment bias [30]. Because weights could only be derived from CRC incidence rates, and not from adenoma or SL rates, the approach was considered suboptimal; therefore, the unweighted model was selected as the primary analysis.

Statistical analyses were conducted using the R software version 4.4.2 (R Core Team). *P* values < 0.05 were considered statistically significant.

## Results

### Characteristics of the study population

Table 1 presents the baseline characteristics of 1816 individuals with LS. Participants completed the FFQ at a median age of 48.8 y (IQR: 33.4–56.2 y), with 58% of the population being female. Most participants were from Australia (48.1%), and the majority had pathogenic variants in either the *MSH2* (42.5%) or *MLH1* (38.1%) gene. During a median follow-up period of 7 y (IQR: 2.8-13.6 y), 742 participants (40.9%) developed a CRN, which included 343 adenomas, 169 SLs, and 230 CRCs.

Total fish intake ranged from 0 to 272.3 g/d, with a median of 13.0 g/d (IQR: 4.5, 21.1 g/d) for males, and from 0 to 113.4 g/d, with a median of 11.4 g/d (IQR: 4.7–19.1 g/d) for females. Compared with participants with the lowest total fish intake, those with higher fish intake were older, more likely to be highly educated, and more likely to have had a history of a CRN diagnosis. They consumed more fruits and vegetables, fiber, and energy. In addition, they were less likely to be *MLH1* carriers and more likely to use NSAIDs/aspirin.

### Total fish intake in relation to CRN risk

**FIGURE 2:**
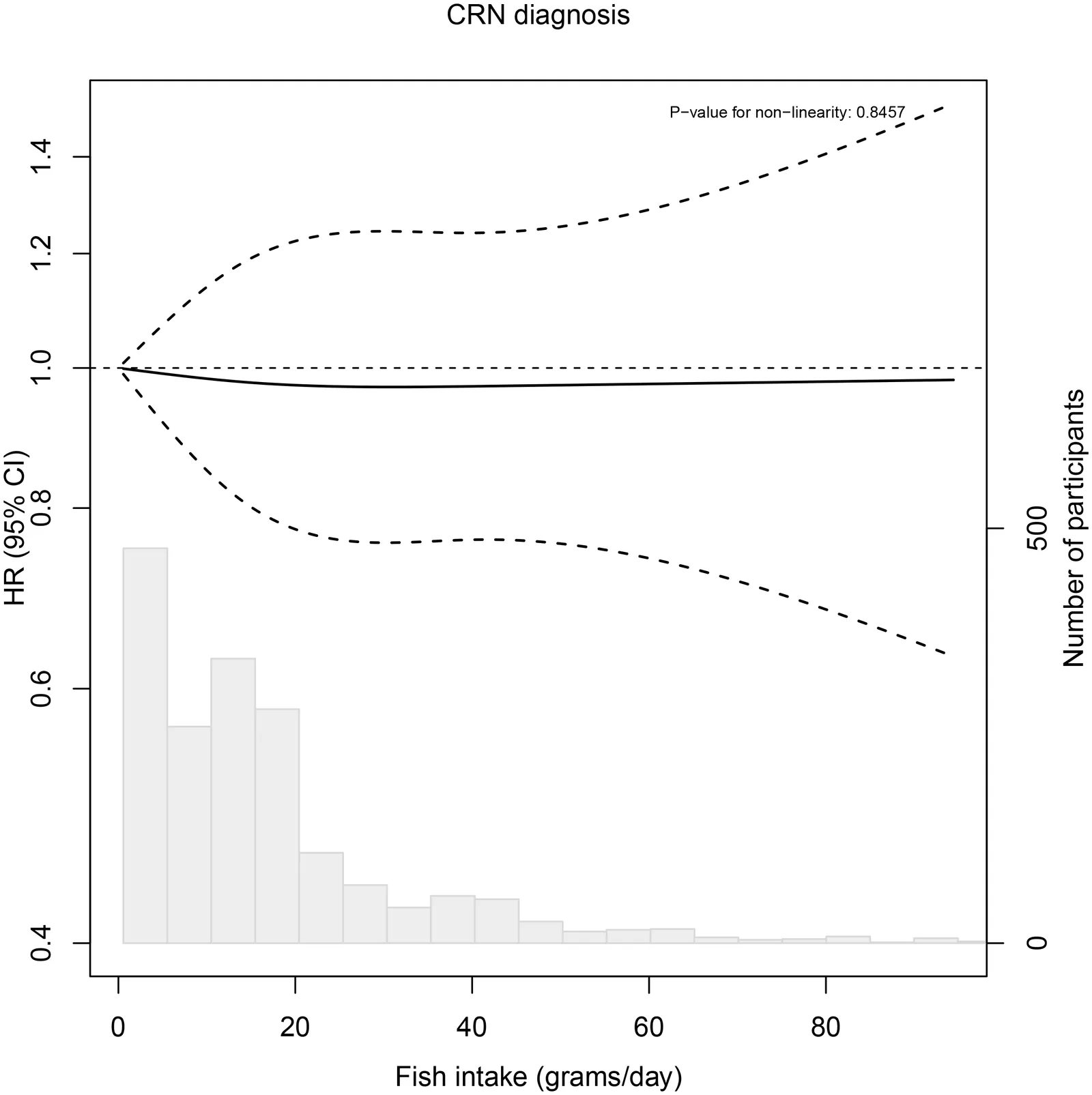
The restricted cubic spline illustrates the association between total fish intake and colorectal neoplasm (CRN) risk, adjusted for age at FFQ completion (continuous), sex, geographical region (Australia, Canada, The Netherlands, United States), education level (low, medium, high), physical activity level (low, medium, high), smoking status (never, former, current), NSAID/aspirin use (no use, less than once per day, at least once per day), alcohol intake (continuous), fruit and vegetables intake (continuous), and red and processed meat intake (continuous). Because of a violation of the proportional hazards assumption, the number of colonoscopies (quartiles) was added as a stratum to the model. The reference value was set at no intake of fish. Knots were located at the 10th, 50th, and 90th percentiles of total fish intake. The graph was truncated at the 1st and 99th percentiles. The *P* value for nonlinearity was 0.85, and the *P* value for association was 0.97. CI, confidence interval; FFQ, food frequency questionnaire; HR, hazard ratio; NSAID, nonsteroidal anti-inflammatory drug.

In the multivariable analyses, no association was observed for the second (Q2), third (Q3), or fourth (Q4) quartile in comparison with the first quartile (Q1) of total fish intake in relation to CRN risk (Table 2). The HR for Q2 compared with Q1 was 0.96 (95% CI: 0.76, 1.23), Q3 compared with Q1 was 0.96 (95% CI: 0.76, 1.22), and Q4 compared with Q1 was 1.03 (95% CI: 0.81, 1.32). For restricted cubic splines, the test for nonlinearity showed no statistically significant result (*P* value: 0.85; Figure 2), suggesting a linear association between total fish intake and CRN risk. The histogram at the bottom of the spline shows that total fish intake was low across the study population. A 10 g daily increase in total fish intake was not associated with CRN risk (HR: 1.00; 95% CI: 0.95, 1.05). All sensitivity analyses yielded similar results (Supplemental Tables 1–5).

**TABLE 2:** Hazard ratio (HR) and 95% confidence intervals (CIs) for total fish intake in relation to colorectal neoplasm risk

	n/events	Events/1000 person-years	Age and sex adjusted model		n/events	Events/1000 person-years	Multivariable model^1^	
			HR	95% CI			HR	95% CI
Total fish intake (g/d)								
Quartiles^2^								
Q1	452/158	37	1.00 (ref)	—	412/143	37	1.00 (ref)	—
Q2	455/176	46	1.14	0.91, 1.44	403/156	48	0.96	0.76, 1.23
Q3	441/216	65	1.55	1.26, 1.90	403/194	64	0.96	0.76, 1.22
Q4	468/192	53	1.24	1.00, 1.53	434/179	54	1.03	0.81, 1.32
Continuous								
Per 10 g/d increment	1816/742	50	1.02	0.99, 1.06	1652/672	50	1.00	0.95, 1.05

Abbreviations: FFQ, food frequency questionnaire; NSAID, nonsteroidal anti-inflammatory drug.

1 Adjusted for age at FFQ completion (continuous), sex, geographical region (Australia, Canada, The Netherlands, United States), education level (low, medium, high), physical activity level (low, medium, high), smoking status (never, former, current), NSAID/aspirin use (no use, less than once per day, at least once per day), alcohol intake (continuous), fruit and vegetables intake (continuous), and red and processed meat intake (continuous). The number of colonoscopies (quartiles) was included as a stratum in the model to account for the violation of the proportional hazards assumption.

2 Sex-specific quartiles (grams per day); males: Q1: <4.5, Q2: 4.5 to <12.9, Q3: 12.9 to <21.2, Q4: ≥21.2. Females: Q1: <4.7, Q2: 4.7 to <11.4, Q3: 11.4 to <19.1, Q4: ≥19.1.

Table 3 presents the HRs, 95% CIs, and *P* values for heterogeneity related to a 10 g daily increase in total fish intake and CRN risk, stratified by NSAID/aspirin use, sex, geographical region, CRN history, pathogenic variant gene type, and cohort. No associations were observed across subgroups.

**TABLE 3:** Hazard ratios (HRs) and 95% confidence intervals (CIs) for a 10 g/d increase of fish intake in relation to colorectal neoplasm (CRN) risk, stratified by NSAID/aspirin use, sex, geographical region, CRN history, type of pathogenic gene variant, or cohort

	*n*/events	Events/ 1000 person-years	Age and sex adjusted model		n/events	Events/ 1000 person-years	Multivariable model^1^		*P*_heterogeneity_^2^
			HR	95% CI			HR	95% CI	
NSAID/aspirin use									
No use	1270/531	49	1.05	1.01, 1.09	1182/497	50	1.01	0.95, 1.07	0.48
Less than once per day	210/121	90	1.03	0.92, 1.15	197/111	87	0.92	0.79, 1.07	
At least once per day	294/74	30	0.92	0.77, 1.09	273/64	28	0.90	0.70, 1.14	
Sex									
Male	759/322	52	1.0	0.95, 1.05	700/299	53	1.01	0.95, 1.08	0.30
Female	1057/420	48	1.06	1.01, 1.12	952/373	47	0.97	0.90, 1.05	
Geographical region^3^									
Australia	874/174	18	1.01	0.94, 1.08	815/162	18	1.01	0.93, 1.11	0.15
Canada	160/58	41	1.10	0.99, 1.22	124/48	45	1.13	0.88, 1.46	
The Netherlands	679/486	147	1.00	0.95, 1.06	619/439	147	0.98	0.92, 1.04	
United States	103/24	30	1.06	0.92, 1.23	94/23	32	1.07	0.95, 1.20	
CRN history									
No CRN history	856/284	34	1.02	0.96, 1.08	781/264	35	0.96	0.88, 1.05	0.87
CRN history	960/458	70	1.02	0.98, 1.06	871/408	68	1.00	0.95, 1.07	
Type of pathogenic gene variant^4^									
*MLH1*	692/282	47	1.04	0.97, 1.11	619/254	48	1.04	0.95, 1.13	0.40
*MSH2/EpCAM*	802/340	53	1.00	0.96, 1.05	730/306	52	0.97	0.91, 1.03	
*MSH6*	240/96	50	1.00	0.88, 1.15	224/88	49	0.92	0.78, 1.09	
*PMS2*	82/24	38	1.08	0.91, 1.29	79/24	41	1.01	0.78, 1.31	
Study cohort									
CCFR	1137/256	22	1.04	0.97, 1.10	1033/233	22	1.02	0.94, 1.10	0.64
GEOLynch	679/486	148	1.00	0.95, 1.06	619/439	147	0.98	0.92, 1.05	

Abbreviations: CCFR, Colon Cancer Family Registry; FFQ, food frequency questionnaire; GEOLynch, Genetic, Environmental and Other factors that influence tumor risk in persons with Lynch syndrome; NSAID, nonsteroidal anti-inflammatory drug.

1 Adjusted for age at FFQ completion (continuous), sex (male, female), education level (low, medium, high), smoking status (never, former, current), BMI 2 y before questionnaire completion (continuous), number of colonoscopies (continuous), alcohol intake (continuous), fruit and vegetable intake (continuous), red and processed meat intake (continuous), physical activity level (low, medium, high), NSAID/aspirin intake (no use, less than once per day, at least once per day) and geographical region. Because the proportional hazards assumption was violated, the number of colonoscopies (quartiles) was added as a stratum to the model.

2 The *P* value for heterogeneity was calculated by using the inverse variance method.

3 Because of violation of the proportional hazards assumption, sex was added as a stratum to the geographical region models.

4 Because of violation of the proportional hazards assumption, smoking status was added as a stratum to the pathogenic gene variant multivariable models.

### Types of fish intake in relation to CRN risk

**FIGURE 3:**
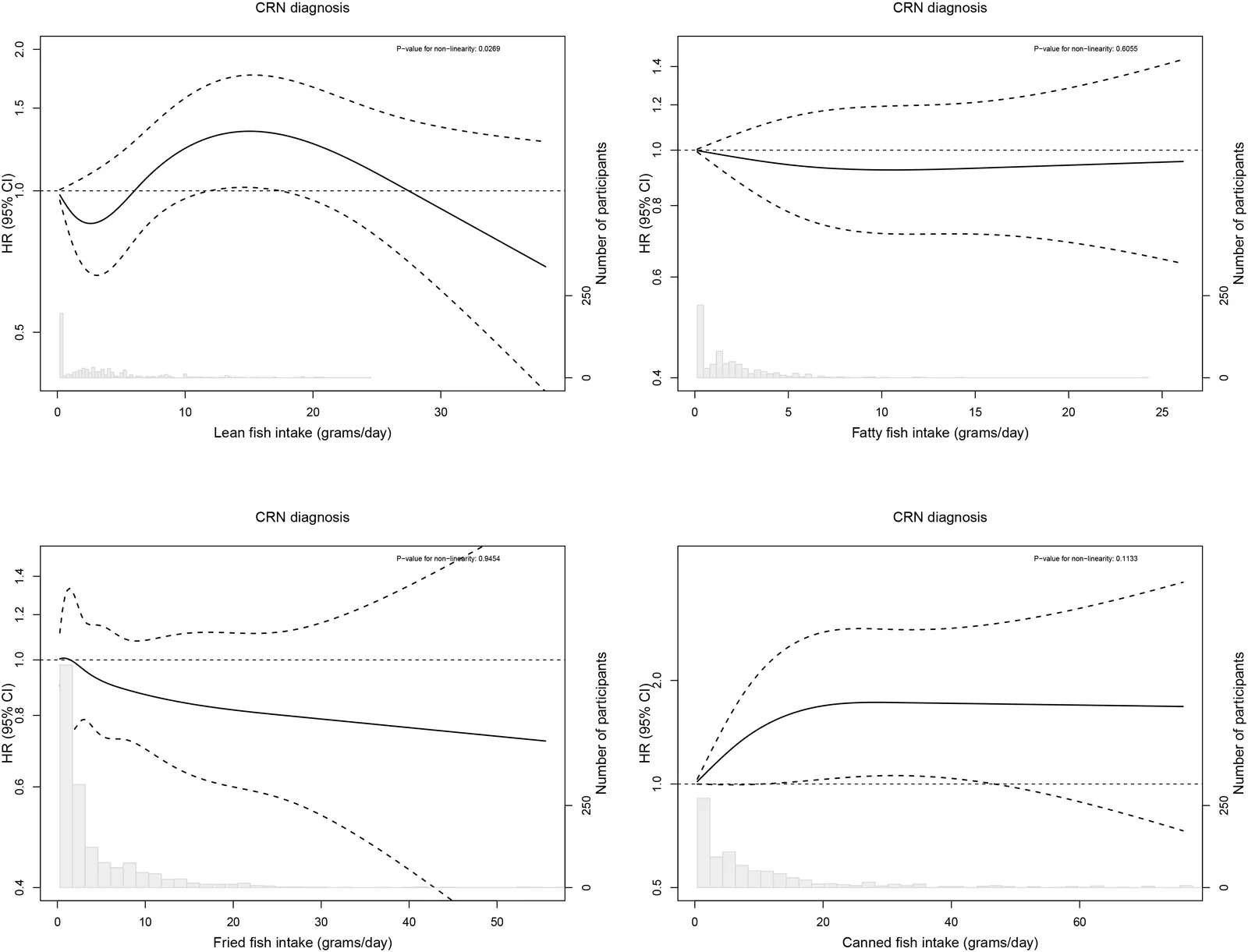
The restricted cubic splines showing the associations between intake of lean fish, fatty fish, fried fish, and canned fish, and the risk of colorectal neoplasm (CRN). Models were adjusted for age at FFQ completion (continuous), sex, geographical region (Australia, Canada, The Netherlands, United States), education level (low, medium, high), physical activity level (low, medium, high), smoking status (never, former, current), NSAID/aspirin use (no use, less than once per day, at least once per day), alcohol intake (continuous), fruit and vegetable intake (continuous), and red and processed meat intake (continuous). Because of a violation of the proportional hazards assumption, the number of colonoscopies (quartiles) was included as a stratum in the model. The reference value was set at no intake for each type of fish. Splines were truncated at the 1st and 99th percentiles. For lean fish, knots were placed at the 5th, 35th, 65th, and 95th percentiles. The HR at 15 g/d vs. 0 g/d was 1.33 (95% CI: 1.02, 1.76); *P* value for nonlinearity: 0.03, *P* value for association: 0.0513. For fatty fish, the knots were placed at the 10th, 50th, and 90th percentiles. The HR per 5 g/d increase was 0.99 (95% CI: 0.91, 1.08); *P* value for nonlinearity: 0.61, *P* value for association: 0.83. For fried fish, the knots were placed at the 5th, 27.5th, 50th, 72.5th, and 95th percentiles. The HR per 5 g/d increase was 0.95 (95% CI: 0.88, 1.04); *P* value for nonlinearity: 0.95, *P* value for association: 0.68. For canned fish, the knots were placed at the 10th, 50th, and 90th percentiles. The HR per 5 g/d increase was 1.06 (95% CI: 1.01, 1.10); *P* value for nonlinearity: 0.11, *P* value for association: 0.09. CI, confidence interval; FFQ, food frequency questionnaire; HR, hazard ratio; NSAID, nonsteroidal anti-inflammatory drug.

Figure 3 displays restricted cubic splines for different types of fish consumption. The histograms accompanying the splines illustrate that intake of the different fish types was low across the study population. The splines showed a nonlinear relationship for lean fish intake (*P* value for nonlinearity 0.03), whereas linear relationships were observed for fatty fish, fried fish, and canned fish (*P* values for nonlinearity 0.61, 0.95, and 0.11, respectively). The overall association for lean fish intake was not statistically significant (*P* value for association: 0.05). However, the highest effect estimate was statistically significant (15 g/d compared with 0 g/d: HR 1.33; 95% CI: 1.02, 1.76). No associations were observed for fatty fish (each 5 g/d increment: HR: 0.99; 95% CI: 0.91, 1.08), and fried fish (each 5 g/d increment: HR: 0.95; 95% CI: 0.88, 1.04), and CRN risk. Although the overall association for canned fish was not statistically significant (*P* value for association: 0.09), a 5 g daily increase in canned fish consumption was associated with a 6% increased risk of developing a CRN (HR: 1.06; 95% CI: 1.01, 1.10).

## Discussion

In this large study of individuals with LS, no association was observed between total fish intake and the risk of CRNs. This null association was consistent across subgroups of NSAID/aspirin use, sex, geographical region, CRN history, MMR pathogenic variant, and study cohort. Analyses of specific fish types revealed no associations for lean, fatty, or fried fish. However, a higher intake of canned fish appeared to be associated with a slightly increased risk of CRNs.

Our finding of no association between total fish intake and CRN risk in individuals with confirmed LS aligns with the results of the retrospective study of Diergaarde et al. [14], who also reported no association with CRN risk in individuals with confirmed and suspected LS [HR for 4.0–<11.9 g/d compared with <4.0 g/d was 1.0 (95% CI: 0.5, 1.9), and ≥11.9 g/d compared with <4.0 g/d was 1.2 (95% CI: 0.6, 2.4)]. Notably, a small subset of participants in that study overlaps with our study population, because it marked the beginning of the GEOLynch cohort, which may partly explain the consistency of findings across both studies. An explanation for our null finding is that heavy metals, such as mercury, lead, and cadmium, as well as organic compounds, such as dioxins and polychlorinated biphenyls, can accumulate in fish [[31], [32], [33], [34]]. These substances have been associated with the development of colorectal polyps [35] and CRC [36,37] and could therefore counteract the potential health benefits of ω-3 fatty acids in fish [38,39]. Overall, our results suggest that total fish intake is unlikely to be associated with the risk of CRNs in individuals with LS.

In contrast, higher intakes of canned fish appeared to be associated with an increased CRN risk. In the restricted cubic splines, the association appeared to have a potential threshold effect at ∼20 g/d. However, intake amounts >20 g/d were low in our study population (*n* = 125), which may have led to instability in the spline estimates at the end of the distribution. To further explore the potential threshold effect, we conducted a post hoc analysis comparing individuals who consumed ≥20 g of canned fish per day with those who consumed <20 g/d. This analysis showed that individuals with ≥20 g/d had an increased risk of CRNs (HR: 1.69; 95% CI: 1.14, 2.50). Although the underlying biological mechanism remains unclear, one plausible explanation is that canned fish contains harmful substances (heavy metals, dioxins, and polychlorinated biphenyls), which can come not only from the bioaccumulation along the aquatic food chain but also from processing [39,40]. The observed association may be related to species-specific bioaccumulation of these harmful substances rather than the canning process itself. Predatory fish, such as tuna, are the most consumed canned fish globally [41] and may contain elevated levels of heavy metals (methylmercury, cadmium, and lead) in their tissues due to bioaccumulation and biomagnification [40,42]. Frequent consumption combined with impaired DNA repair capacity in LS may result in more pronounced effects in individuals with LS than in the general population, potentially explaining the increased risk observed in our study. However, in our study, no information was available on the specific species of canned fish consumed. To date, only 1 study has investigated the association between canned fish and CRC risk in the general population. This study combined data from 2 case-control studies and reported an inverse association [43]. Notably, the study specifically investigated canned fish preserved in olive oil, whereas in our study, information on the packing medium (oil compared with water) was unavailable. Altogether, these contrasting findings highlight the need for further research to clarify the role of canned fish consumption and CRN risk, especially among individuals with LS.

This study is the largest to date examining the association between fish intake and CRN risk in individuals with LS, including individuals with confirmed LS from multiple countries, such as Australia, the United States, Canada, and the Netherlands, with a substantial number of CRN events developing during follow-up. Furthermore, this is the first study to explore associations between various types of fish and CRN risk in LS carriers. Reverse causality is unlikely, as analyses from the GEOLynch study showed no evidence that individuals with LS change their dietary habits after a CRN diagnosis [44]. This was supported by our sensitivity analysis, excluding individuals diagnosed within 100 d of completing the FFQ, and by stratified analyses by CRN history, which yielded similar results. Another strength of this study is the adjustment for multiple potential confounders. Among these, geographical region had the largest impact on the association, possibly due to differences in follow-up duration, the number of CRN events, follow-up procedures, and questionnaires.

A limitation of this study is that overall fish consumption, both total and by type, was low in this population, which may have reduced our ability to detect associations. Nevertheless, this likely reflects typical dietary intake in daily practice. No additional data, such as plasma ω-3 fatty acid concentrations and fish oil supplement intake, were available in this large population to evaluate the association between fish intake and CRN risk in LS carriers in more depth. However, in a previous analysis from the GEOLynch study, we did not find an association between fish oil supplement use and adenoma risk in individuals with LS [45].

In conclusion, our findings suggest that total fish intake is not associated with the risk of CRNs in individuals with LS overall, nor in subgroups defined by NSAIDs/aspirin use, sex, geographical region, CRN history, pathogenic MMR variant, or cohort. No associations were found for lean fish, fatty fish, and fried fish intake, whereas a higher consumption of canned fish may be associated with an increased CRN risk. These results underscore the need for further research to clarify the role of canned fish in CRN risk.

## Consent to participate

Participants gave written informed consent to investigators to collect and share study data (including self-reported data, clinical records, cancer registry data, molecular test result data, and biospecimens), if any.

## Author contributions

The author’s responsibilities were as follows – MTB, MEvL, PAN, EK, FJBvD: contributed to conceptualization; MTB, EW, HJMS-W, EK, FJBvD: contributed to methodology; MTB: contributed to formal analysis, investigation, data curation, visualization, writing – original draft preparation; MTB, FJBvD: contributed to project administration; MTB, EW, TMB, JJK, MEvL, LLM, PAN, NJS, HJMS-W, EK, FJBvD: contributed to writing – review and editing; MEvL, LLM, PAN, NJS: contributed to resources; MEvL, PAN, EK, FJBvD: contributed to funding acquisition, EK, FJBvD: contributed to supervision, and all authors: read and approved the final manuscript.

## Ethics approval

Local medical ethics review committees approved both studies: GEOLynch and the Colon Cancer Family Registry. GEOLynch was approved by the Committee on Research Involving Human Subjects, region Arnhem-Nijmegen (CMO-nr: 2005/283). The CCFR study protocols were approved by each CCFR site’s Human Subjects/Ethics boards: Cleveland Clinic Institutional Review Board file nr: 2913; University of Hawaii Human Studies Program file nr: 10057; University of California, San Francisco Human Research Protection Program Institutional Review Board file nr: 17-23404; Mount Sinai Hospital Research Ethics Board file nr: 01-0347-U, and; University of Melbourne Health Sciences Human Ethics Sub-Committee Ethics ID nr: 13094.

## Data availability

The data that support the findings of this study are available on request from the corresponding author. The data are not publicly available due to privacy or ethical restrictions. The Colon Cancer Family Registry (CCFR) investigators and institutions affirm their intention to share research data consistent with all relevant NIH resource/data sharing policies. The CCFR data reported in this manuscript can be requested by contacting the corresponding author and submitting an Application for Collaboration to the CCFR (https://coloncfr.org/for-researchers/collaborate-with-the-ccfr/).

## Funding

The GEOLynch study was financially supported by the Dutch Cancer Society (grant UW-2005-3275 and KUN-2007-3842), Wereld Kanker Onderzoek Fonds (WKOF) & World Cancer Research Fund International (WCRF International), as well as by funds from grant 2014/1184 and IIG_FULL_2021_024 as part of the WCRF International Grant Program. The Colon Cancer Family Registry (CCFR, www.coloncfr.org) is supported in part by funding from the National Cancer Institute (NCI), NIH (award U01 CA167551). Support for case ascertainment was provided in part from the Surveillance, Epidemiology, and End Results Program and the following United States’ state cancer registries: AZ, CO, MN, NC, NH; and by the Victoria Cancer Registry (Australia) and Ontario Cancer Registry (Canada). The funding sources had no role in the study design; in the collection, analysis, and interpretation of data; in the writing of the report; and in the decision to submit the article for publication. This manuscript is the result of funding in whole or in part by the NIH. It is subject to the NIH Public Access Policy. Through acceptance of this federal funding, NIH has been given the right to make this manuscript publicly available in PubMed Central upon the Official Date of Publication, as defined by NIH.

## Declaration of generative AI and AI-assisted technologies in the writing process

The author(s) declare that no generative AI or AI-assisted technologies were used in the writing of this manuscript.

## Conflict of interest

The authors report no conflicts of interest.
