# Supplementary material for: Fish Consumption and Colorectal Neoplasms among Lynch Syndrome Carriers
Source: J Nutr. 2026 Jun 17;156(8):101670. doi: 10.1016/j.tjnut.2026.101670 (PMC13494212; doi:10.1016/j.tjnut.2026.101670)
Supplement: Supplementary file 1 [file mmc1.docx]

**Supplementary Material**

**Fish consumption and colorectal neoplasms among Lynch syndrome carriers**

**Short title:** Fish and Lynch syndrome

**Marjolein T. Bulbaai^1^**

1 Division of Human Nutrition and Health, Wageningen University & Research, Wageningen, the Netherlands.

**Correspondence**

Marjolein T. Bulbaai; Stippeneng 4, 6708 WE Wageningen, the Netherlands; [marjolein.bulbaai@wur.nl](mailto:marjolein.bulbaai@wur.nl) ; +31 317487424

Fränzel J.B. van Duijnhoven; Stippeneng 4, 6708 WE Wageningen, the Netherlands; franzel.vanduijnhoven@wur.nl; +31 317485375

| **Table S1.** Sensitivity analysis excluding participants who had a CRN within 100 days after FFQ completion | | | | | | | | |
| --- | --- | --- | --- | --- | --- | --- | --- | --- |
|  | n/Events | Events/ 1000 person-years | Age and sex adjusted model |  | n/Events | Events/ 1000 person-years | Multivariable model^a^ |  |
|  |  |  | HR | 95% CI |  |  | HR | 95% CI |
| **Total fish intake (g/day)** |  |  |  |  |  |  |  |  |
| **Quartiles**^b^ |  | | | | | | | |
| Q1 | 445/151 | 36 | 1.00 (ref) | - | 405/136 | 35 | 1.00 (ref) | - |
| Q2 | 442/163 | 43 | 1.11 | 0.87, 1.41 | 390/143 | 44 | 0.93 | 0.73, 1.20 |
| Q3 | 431/206 | 62 | 1.56 | 1.26, 1.93 | 395/186 | 61 | 0.98 | 0.77, 1.25 |
| Q4 | 458/182 | 50 | 1.23 | 0.99, 1.54 | 425/170 | 51 | 1.04 | 0.81, 1.34 |
| **Continuous** |  | | | | | | | |
| Per 10 g/day increment | 1776/702 | 47 | 1.03 | 0.99, 1.07 | 1615/635 | 47 | 1.00 | 0.95, 1.06 |
| ^a^Adjusted for age at FFQ completion (continuous), sex, geographic region (Australia, Canada, the Netherlands, USA), education level (low, medium, high), physical activity level (low, medium, high), smoking status (never, former, current), NSAID use (no use, less than once per day, at least once per day), alcohol intake (continuous), fruit and vegetables intake (continuous), and red and processed meat intake (continuous). The number of colonoscopies (quartiles) was included as a stratum in the model to account for the violation of the proportional hazards assumption.  ^b^ Sex-specific quartiles (grams per day); males: Q1: <4.5, Q2: 4.5- <12.9, Q3: 12.9-<21.2, Q4: ≥21.2. Females: Q1: <4.7, Q2: 4.7- <11.4, Q3: 11.4-<19.1, Q4: ≥19.1. | | | | | | | | |

**Tables of sensitivity analyses (Table S1-S5)**

| **Table S2.** Sensitivity analysis with adenoma as the outcome | | | | | | | | |
| --- | --- | --- | --- | --- | --- | --- | --- | --- |
|  | n/Events | Events/ 1000 person-years | Age and sex adjusted model |  | n/Events | Events/ 1000 person-years | Multivariable model^a^ |  |
|  |  |  | HR | 95% CI |  |  | HR | 95% CI |
| **Total fish intake (g/day)** |  |  |  |  |  |  |  |  |
| **Quartiles**^b^ |  | | | | | | | |
| Q1 | 452/86 | 20 | 1.00 (ref) | - | 412/76 | 19 | 1.00 (ref) | - |
| Q2 | 455/101 | 25 | 1.17 | 0.87, 1.58 | 403/86 | 25 | 0.80 | 0.58, 1.11 |
| Q3 | 441/148 | 42 | 1.86 | 1.44, 2.41 | 403/131 | 41 | 0.89 | 0.67, 1.20 |
| Q4 | 468/118 | 31 | 1.36 | 1.02, 1.82 | 434/107 | 31 | 0.98 | 0.71, 1.37 |
| **Continuous** |  | | | | | | | |
| Per 10 g/day increment | 1816/453 | 29 | 1.05 | 1.0, 1.09 | 1652/400 | 28 | 1.01 | 0.94, 1.08 |
| ^a^Adjusted for age at FFQ completion (continuous), sex, geographic region (Australia, Canada, the Netherlands, USA), education level (low, medium, high), physical activity level (low, medium, high), smoking status (never, former, current), NSAID use (no use, less than once per day, at least once per day), alcohol intake (continuous), fruit and vegetables intake (continuous), and red and processed meat intake (continuous). The number of colonoscopies (quartiles) was included as a stratum in the model to account for the violation of the proportional hazards assumption.  ^b^ Sex-specific quartiles (grams per day); males: Q1: <4.5, Q2: 4.5- <12.9, Q3: 12.9-<21.2, Q4: ≥21.2. Females: Q1: <4.7, Q2: 4.7- <11.4, Q3: 11.4-<19.1, Q4: ≥19.1. | | | | | | | | |

| **Table S3.** Sensitivity analysis with CRC as the outcome | | | | | | | | |
| --- | --- | --- | --- | --- | --- | --- | --- | --- |
|  | n/Events | Events/ 1000 person-years | Age and sex adjusted model |  | n/Events | Events/ 1000 person-years | Multivariable model^a^ |  |
|  |  |  | HR | 95% CI |  |  | HR | 95% CI |
| **Total fish intake (g/day)** |  |  |  |  |  |  |  |  |
| **Quartiles**^b^ |  | | | | | | | |
| Q1 | 452/72 | 15 | 1.00 (ref) | - | 412/67 | 15 | 1.00 (ref) | - |
| Q2 | 455/68 | 15 | 0.93 | 0.65, 1.31 | 403/62 | 15 | 0.93 | 0.64, 1.36 |
| Q3 | 442/71 | 16 | 0.95 | 0.69, 1.32 | 404/66 | 16 | 1.01 | 0.70, 1.44 |
| Q4 | 468/78 | 17 | 1.02 | 0.75, 1.39 | 434/75 | 18 | 1.05 | 0.75, 1.48 |
| **Continuous** |  | | | | | | | |
| Per 10 g/day increment | 1817/289 | 16 | 1.01 | 0.95, 1.08 | 1653/270 | 16 | 1.02 | 0.95, 1.09 |
| ^a^Adjusted for age at FFQ completion (continuous), sex, geographic region (Australia, Canada, the Netherlands, USA), education level (low, medium, high), physical activity level (low, medium, high), smoking status (never, former, current), NSAID use (no use, less than once per day, at least once per day), alcohol intake (continuous), fruit and vegetables intake (continuous), and red and processed meat intake (continuous). The number of colonoscopies (quartiles) was included as a stratum in the model to account for the violation of the proportional hazards assumption.  ^b^ Sex-specific quartiles (grams per day); males: Q1: <4.5, Q2: 4.5- <12.9, Q3: 12.9-<21.2, Q4: ≥21.2. Females: Q1: <4.7, Q2: 4.7- <11.4, Q3: 11.4-<19.1, Q4: ≥19.1. | | | | | | | | |

| **Table S4.** Sensitivity analysis with advanced CRN as the outcome | | | | | | | | |
| --- | --- | --- | --- | --- | --- | --- | --- | --- |
|  | n/Events | Events/ 1000 person-years | Age and sex adjusted model |  | n/Events | Events/ 1000 person-years | Multivariable model^a^ |  |
|  |  |  | HR | 95% CI |  |  | HR | 95% CI |
| **Total fish intake (g/day)** |  |  |  |  |  |  |  |  |
| **Quartiles**^b^ |  | | | | | | | |
| Q1 | 452/102 | 22 | 1.00 (ref) | - | 412/92 | 22 | 1.00 (ref) | - |
| Q2 | 455/107 | 25 | 1.05 | 0.78, 1.40 | 403/97 | 26 | 0.96 | 0.69, 1.32 |
| Q3 | 441/136 | 34 | 1.36 | 1.05, 1.76 | 403/123 | 34 | 1.08 | 0.81, 1.43 |
| Q4 | 468/117 | 28 | 1.09 | 0.84, 1.41 | 434/111 | 29 | 1.03 | 0.78, 1.37 |
| **Continuous** |  | | | | | | | |
| Per 10 g/day increment | 1816/462 | 27 | 1.02 | 0.97, 1.07 | 1652/423 | 27 | 1.01 | 0.96, 1.07 |
| ^a^Adjusted for age at FFQ completion (continuous), sex, geographic region (Australia, Canada, the Netherlands, USA), education level (low, medium, high), physical activity level (low, medium, high), smoking status (never, former, current), NSAID use (no use, less than once per day, at least once per day), alcohol intake (continuous), fruit and vegetables intake (continuous), and red and processed meat intake (continuous). The number of colonoscopies (quartiles) was included as a stratum in the model to account for the violation of the proportional hazards assumption.  ^b^ Sex-specific quartiles (grams per day); males: Q1: <4.5, Q2: 4.5- <12.9, Q3: 12.9-<21.2, Q4: ≥21.2. Females: Q1: <4.7, Q2: 4.7- <11.4, Q3: 11.4-<19.1, Q4: ≥19.1. | | | | | | | | |

| **Table S5.** Sensitivity analysis: Weighted cohort method | | | | | | | | |
| --- | --- | --- | --- | --- | --- | --- | --- | --- |
|  | n/Events | Events/ 1000 person-years | Age and sex adjusted model |  | n/Events | Events/ 1000 person-years | Multivariable model^a^ |  |
|  |  |  | HR | 95% CI |  |  | HR | 95% CI |
| **Total fish intake (g/day)** |  |  |  |  |  |  |  |  |
| **Quartiles**^b^ |  | | | | | | | |
| Q1 | 452/158 | 37 | 1.00 (ref) | - | 412/143 | 37 | 1.00 (ref) | - |
| Q2 | 455/176 | 46 | 1.20 | 0.90, 1.60 | 403/156 | 48 | 1.00 | 0.74, 1.35 |
| Q3 | 441/216 | 65 | 1.61 | 1.23, 2.10 | 403/194 | 64 | 0.99 | 0.74, 1.33 |
| Q4 | 468/192 | 53 | 1.28 | 0.99, 1.66 | 434/179 | 54 | 1.15 | 0.85, 1.57 |
| **Continuous** |  | | | | | | | |
| Per 10 g/day increment | 1816/742 | 50 | 1.03 | 0.99, 1.07 | 1652/672 | 50 | 1.01 | 0.96, 1.07 |
| ^a^Adjusted for age at FFQ completion (continuous), sex, geographic region (Australia, Canada, the Netherlands, USA), education level (low, medium, high), physical activity level (low, medium, high), smoking status (never, former, current), NSAID use (no use, less than once per day, at least once per day), alcohol intake (continuous), fruit and vegetables intake (continuous), and red and processed meat intake (continuous). The number of colonoscopies (quartiles) was included as a stratum in the model to account for the violation of the proportional hazards assumption.  ^b^ Sex-specific quartiles (grams per day); males: Q1: <4.5, Q2: 4.5- <12.9, Q3: 12.9-<21.2, Q4: ≥21.2. Females: Q1: <4.7, Q2: 4.7- <11.4, Q3: 11.4-<19.1, Q4: ≥19.1. | | | | | | | | |
